# *Stenotrophomonas maltophilia* neonatal sepsis: a case report

**DOI:** 10.1186/s13256-024-04479-2

**Published:** 2024-03-25

**Authors:** Williams Oluwatosin Adefila, Isaac Osie, Modou Lamin Keita, Baleng Mahama Wutor, Abdulsalam Olawale Yusuf, Ilias Hossain, Minteh Molfa, Ousman Barjo, Rasheed Salaudeen, Grant Mackenzie

**Affiliations:** 1 Medical Research Council Unit The Gambia at London, School of Hygiene and Tropical Medicine, West Africa, PO Box 273, Fajara, The Gambia; 2 Faculty of Infectious and Tropical Diseases, London School of Hygiene and Tropical Medicine, London, UK; 3 Murdoch Children’s Research Institute, Melbourne, Australia; 4 Department of Paediatrics, University of Melbourne, Melbourne, Australia

**Keywords:** Case report, Neonatal sepsis, *Stenotrophomonas maltophilia*, Antibiotic treatment, Multidrug-resistant

## Abstract

**Background:**

*Stenotrophomonas maltophilia* is a gram-negative bacteria known for causing opportunistic and nosocomial infections in humans. *S. maltophilia* is an emerging pathogen of concern due to it’s increasing prevalence, diverse disease spectrum, intrinsic multi-drug resistance and high mortality rates in immunocompromised individuals. S. *maltophilia* is a rare cause of neonatal sepsis associated with significant morbidity and mortality. The bacterium’s multi-drug resistance poses a considerable challenge for treatment, with various mechanisms contributing to its resistance.

**Case presentation:**

We report a case involving a 40-h-old male African neonate who exhibited symptoms of neonatal sepsis. The blood culture revealed *Stenotrophomonas maltophilia*, which was sensitive to ciprofloxacin and gentamicin but resistant to other antibiotics. Lumbar puncture for CSF could not be done because the father declined.

We treated the newborn with the empirical first-line antibiotics as per the national guideline intravenous ampicillin and gentamicin for six days, and the child recovered fully with a repeated negative blood culture.

**Conclusions:**

This report describes a neonatal sepsis case caused by *S. maltophilia,* a multi-drug resistant bacteria and a rare cause of neonatal sepsis. We report that early detection of the bacterial and antimicrobial management based on local antibiogram data may be essential for successful patient’s management.

## Background

*Stenotrophomonas maltophilia* causes human infections ranging from bacteremia, sepsis, endocarditis, pneumonia, and meningitis. It is a gram-negative, glucose non-fermentative aerobic rod with low virulence. The bacteria was described in 1961 by Hugh and Ryschenkow [1].

*S. maltophilia* is often pathogenic in immunocompromised and hospitalized patients, especially in the intensive care unit (ICU) [2]. It can also produce biofilms on prosthetic devices, such as catheters, mechanical ventilators, and feeding tubes [3]. The mortality rate of an *S. maltophilia* infection is high, primarily when associated with pneumonia (or bacteremia) and antibiotic resistance [4]. The resistance can be intrinsic, or the bacteria can develop resistance to antibiotics over time. The increasing use of antibiotics, especially broad-spectrum antibiotics, has contributed to the rise of multidrug-resistant (MDR) strains of *S. maltophilia*. MDR strains are resistant to a wide range of antibiotics, making them difficult to treat [5]. The outbreak of SARS-CoV-2 may have contributed to the increase in the prevalence of MDR bacteria, including *S. maltophilia*, because of the increased irrational use of antibiotics to treat COVID-19 [6].

## Case presentation

A 40-h-old male African neonate was brought by the mother to our health facility, Bansang Hospital, at 7:30 am. Bansang Hospital (a secondary care facility) is in rural Gambia. It is the main referral centre for the country’s two largest regions (Central and Upper River Region). The mother complained of a persistently high fever that started about a day after delivery. She also complained of the child’s refusal to breastfeed with poor suckling ability, and was highly irritable. The mother also complained that the child had multiple episodic jerky movements, clenching the fist, lip-smacking, upward eye rolling, and limb stiffness during each episode. She noticed that the child had abnormal rapid breathing and a gradual decline in the child’s alertness. There was no history of vomiting or passage of loose, watery stool. There was no history of yellowish discolouration of the eye or the skin.

The mother was a primigravid, carrying the pregnancy to term with only two antenatal visits. She was not a known diabetic or hypertensive. She was not on any long-term medication. She was HIV-negative and received the required prenatal tetanus toxoid vaccination.

The pregnancy was carried to term. There was a history of premature membrane rupture and liquor drainage for more than two days before the onset of labour. The draining amniotic fluid was not blood-stained but had a foul odour. The mother also complained of a foul-smelling whitish-brown vaginal discharge with no associated itching two weeks before the delivery. The mother did not receive any antibiotics before delivery. She delivered in the hospital through spontaneous vaginal delivery after more than 18 h of prolonged labour. The child’s birth weight was 3kg, and the newborn did not cry immediately after birth, necessitating further resuscitation. Apgar score in the first minute was three, and at ten minutes, it was seven. After a successful resuscitation, the child was allowed to breastfeed, and the mother described the suckling reflex as weak. They were discharged home less than 24 h after delivery.

The parents noticed the newborn having a persistent high-grade fever that started the following day after birth, not feeding, and becoming weak at home. The grandmother gave the newborn some undetermined syrups to reduce the fever. The newborn was not improving; hence, the mother brought him to the hospital.

Physical examination revealed an acutely ill-looking newborn with hyperpyrexia (temperature of 39.1 degrees Celsius), not pale, anicteric, not cyanosed, responsive to pain only and having seizures. The child weighed 3kg.

The central nervous system examination showed a newborn with poor suckling reflex, unresponsive except to pain, poor Moro reflex, normal fontanelles, normal tones globally and lethargic.

The respiratory rate was 65 breaths per minute, hypoxia with oxygen saturation of 80%, absent subcostal retraction, nil dullness to chest percussion, and broncho-vesicular breath sounds.

His heart rate was 153 beats per minute, with no abnormal heart sounds on auscultation.

The rest of the physical examination was unremarkable.

The random blood sugar was 5.1 mmol/l (91.8 mg/dl). We requested a blood culture, microscopy, and sensitivity, and a chest x-ray.

We counselled the parents for lumbar puncture for cerebrospinal fluid analysis, but the father declined.

A diagnosis of early-onset neonatal sepsis with a differential diagnosis of neonatal meningitis and perinatal asphyxia was made.

The newborn was started on empirical first-line antibiotics as per the national guideline: IV ampicillin 50mg/kg every 6 h and IV gentamicin 5mg/kg daily. Also, we put the child on nil per oral, intravenous maintenance fluid 34mls every three hours and a stat dose of IV phenobarbitone. Moreover, we placed the child on humidified oxygen, one litre per minute, through a nasal prong and monitored the vital signs, including random blood sugar.

### Results of investigation

The chest x-ray findings were essentially normal. The blood culture yielded glucose non-fermenting colonies, and the Gram stain showed gram-negative rods. We processed the bacterium for identification using the Analytical Profile Index (API) 20E. The identification number 1202000 on the API is consistent with *Stenotrophomonas maltophilia.* Antibiotic susceptibility testing of the pathogen using the disc diffusion method showed sensitivity to ciprofloxacin and gentamicin. The organism was resistant to ceftriaxone, tetracycline, chloramphenicol, and ampicillin.

### Progress report

After 48 h of admission, the fever subsided, and the child had one episode of convulsion. He was alert but had a poor suckling reflex. After 72 h of hospital admission, he had no seizures in the previous 36 h, had started breastfeeding, and no complaints or abnormalities were noted. After four days of hospital admission, he had a normal suckling reflex, and the mother had adequate lactation. No anomaly was detected, and we stopped the intravenous maintenance fluid. We did not change the antibiotic regimen because the child improved well clinically on the initial antibiotics administered. There were no observed adverse effects or complications during the treatment. After six days on admission, the newborn was discharged home for a follow-up in one week. The mother had no complaints at follow-up, and the child was feeding well with no abnormal signs.

## Discussion and conclusions

The prevalence of *Stenotrophomonas maltophilia* isolation described from different hospitals ranged from 7.1 to 37.7 cases per 10,000 discharges or 18.3 cases per 1,000 patient days. The majority of these reports were investigated after a perceived increase in the incidence and prevalence of the organism[7].

Neonatal sepsis is a leading cause of death in newborns in sub-Saharan Africa. It is estimated that 380,000 to 2 million cases of neonatal sepsis occur in sub-Saharan Africa each year and that 270,000 are fatal [7]. Neonatal resuscitation, low birth weight, and prematurity are risk factors for neonatal sepsis. Prolonged and premature membrane rupture, multiple vaginal examinations, meconium-stained amniotic fluid, and intrapartum fever are maternal risk factors for neonatal sepsis [8]. Gram-positive bacteria *(Staphylococcus aureus*, *Streptococcus pyogenes,* group B *streptococcus,* and group D *streptococcus*) and gram-negative bacteria (*Klebsiella, Escherichia coli, Pseudomonas, Enterobacter spp,* and *Salmonella* spp.) are the most common causes of neonatal sepsis in sub-Saharan Africa [9–11].

*S*. *maltophilia* is a rare cause of neonatal sepsis globally and in sub-Saharan Africa, but its emergence has significant implications for patient management due to antimicrobial resistance [12]. *S. maltophilia* is categorized by the World Health Organization (WHO) categorized as a primary multi-drug-resistant bacteria in hospital settings [13]. The prevalence of *S. maltophilia* infections increased in the general population, with more cases in the Western Pacific regions (from 6% to 18.6%) and lower prevalence in the American regions (from 3.2% to 5.7%) in 1991 and 2019 [13]. *S. maltophilia* has exhibited an increasing tendency towards drug resistance through multiple mechanisms. These mechanisms include the expression of resistance genes acquired from the environment, reduced outer membrane permeability, and transposons encoded within the chromosome and plasmids [14]. Furthermore, other mechanisms of antimicrobial resistance include the production of β-lactamase enzymes, integrons, biofilms, and multi-drug efflux [15–17].

Our patient’s mother had prolonged prelabour rupture of membranes and prolonged labour. These risk factors predisposed the neonate to acquire sepsis with *S. maltophilia.* Also, the neonate had perinatal asphyxia and needed to be resuscitated. This was another avenue through which the neonate may have acquired the infection.

Even though prematurity and low birth weight have been reported as risk factors for *S. maltophilia* infection in the newborn*,* our case was term and had a normal birth weight but with significant other risk factors for sepsis [17, 18]. Since 1977, the first case of *S. maltophilia* was documented, and only very few cases of meningitis associated with S. maltophilia have been documented [19, 20]. The management of *S. maltophilia* infection is challenging due to the bacterium’s resistance to multiple antimicrobials used to treat Gram-negative infections [10]. Trimethoprim-sulfamethoxazole and flouroquininolones are typically effective against *S. maltophilia* strains, but due to its unfavourable side effects, it is not recommended for use in newborns [21]. In our patient, we found that the pathogen was susceptible to ciprofloxacin and gentamicin and resistant to ceftriaxone, tetracycline, chloramphenicol, and ampicillin. The organism’s resistance to commonly available antibiotics such as ampicillin and ceftriaxone, poses a challenge to effective clinical management. It increases the chances of neonatal mortality and morbidity, especially in resource-limited settings.

Neonatal sepsis can be devastating due to its high mortality and potential long-term consequences and the risk is further amplified when *S. maltophilia* is indicated [22]. However, this can be prevented through early detection. Early detection of *S. maltophilia* and management is critical because of its aggressive nature as an opportunistic pathogen, non-specific neonatal symptoms, rapid progression, and improved treatment outcomes [23].

The index newborn responded well to the first-line empirical treatment combination of ampicillin and gentamicin, and the child recovered fully. Hence, it is essential to note that the chosen antibiotic combination had a high success rate despite the organism’s inherent multi-drug resistance. Furthermore, this case has highlighted the role of *S. maltophilia* as an emerging cause of nosocomial infections in vulnerable patients such as neonates in resource limited settings. Clinicians should have a high suspicion to identify patients at high risk for *S. maltophilia* infections. Early detection and prompt management using clinical and microbiological evidence are paramount to successfully managing *S. maltophilia* neonatal sepsis.

Due to the parents’ refusal to perform a lumbar puncture to confirm our suspicion of meningitis, a blood culture was essential for diagnosing *S. maltophilia* infection in our patient. We had other limitations in conducting further crucial diagnostic investigations like complete blood count and C-reactive protein because our laboratory is under-equipped.

This report shows that although *S. maltophilia* is a rare cause of neonatal sepsis, early detection and management based on local antibiogram data is essential for excellent patient outcomes. The lack of microbiology services in many settings in sub-Saharan Africa will mean that infections with *S. maltophilia* are under-detected and often inadequately treated.

## Data Availability

Data sharing is not applicable to this article as no datasets were generated or analyzed during the current study.

## Abbreviations

IV: Intravenous
ICU: Intensive care unit
MDR: Multi-drug resistant
API: Analytical Profile Index
WHO: World Health Organization
